# Channel Measurement and Modeling for 5G Urban Microcellular Scenarios

**DOI:** 10.3390/s16081330

**Published:** 2016-08-20

**Authors:** Michael Peter, Richard J. Weiler, Barış Göktepe, Wilhelm Keusgen, Kei Sakaguchi

**Affiliations:** 1 Department of Wireless Communications and Networks, Fraunhofer Heinrich Hertz Institute, Berlin 10587, Germany; richard.weiler@hhi.fraunhofer.de (R.J.W.); baris.goektepe@hhi.fraunhofer.de (B.G.); wilhelm.keusgen@hhi.fraunhofer.de (W.K.); kei.sakaguchi@hhi.fraunhofer.de (K.S.); 2 Department of Electrical and Electronic Engineering, Tokyo Institute of Technology, Tokyo 152-8552, Japan

**Keywords:** millimeter-wave propagation, channel sounding, urban micro, 60 GHz channel, 5G channel model, ray tracing

## Abstract

In order to support the development of channel models for higher frequency bands, multiple urban microcellular measurement campaigns have been carried out in Berlin, Germany, at 60 and 10 GHz. In this paper, the collected data is uniformly analyzed with focus on the path loss (PL) and the delay spread (DS). It reveals that the ground reflection has a dominant impact on the fading behavior. For line-of-sight conditions, the PL exponents are close to free space propagation at 60 GHz, but slightly smaller (1.62) for the street canyon at 10 GHz. The DS shows a clear dependence on the scenario (median values between 16 and 38 ns) and a strong distance dependence for the open square and the wide street canyon. The dependence is less distinct for the narrow street canyon with residential buildings. This behavior is consistent with complementary ray tracing simulations, though the simplified model tends to overestimate the DS.

## 1. Introduction

Fifth generation (5G) mobile networks will need to make use of frequencies above 6 GHz to provide multi-Gbps data rates and realize ultra-high capacity for various deployment scenarios [1,2,3]. The targeted frequency bands range up to 100 GHz [4]. Early stage studies have shown the feasibility of millimeter waves for cellular networks [5,6,7], and standardization activities for 5G have been kicked off in the Radiocommunication Sector of the International Telecommunication Union (ITU-R) [8] and the 3rd Generation Partnership Project (3GPP) [9]. Considerable effort is currently put into the refinement of channel models, since they are essential for accurately assessing the performance of future deployments. Most relevant work builds upon state-of-the-art three-dimensional (3D) geometry-based stochastic channel models (GSCMs) that have been developed recently for lower frequency bands. New model features and extended parameter tables have been proposed to fulfill the requirements for 5G millimeter-wave (mm-wave) channel models, which have to support a much wider frequency range, large antenna arrays, large bandwidth and high mobility [10,11,12,13,14,15].

Providing measurement data for their validation that is both statistically reliable [16] and including full directional information is very challenging as long as high performance electronically steerable antennas for various mm-wave frequency bands are not available at reasonable expense. Multiple channel sounding campaigns have been reported in the literature for cellular access scenarios above 10 GHz, e.g., at 11 GHz [17], 28 GHz [18,19], 38 and 73 GHz [20] and 60 GHz [21]. The amount of available data, however, is still not sufficient to fully substantiate and reliably parameterize the models. Currently, directional information is mainly obtained by successively scanning all (relevant) angles with mechanically steerable directional antennas [21]. Since the scanning takes a long time, the number of observation points is limited in practice. A different measurement approach, which is also pursued in this work, is to use a channel sounder with a high measurement repetition rate in conjunction with omnidirectional antennas and a mobile receiver (RX). It allows for collecting large data sets and capturing all multipaths even for time-variant channels and aims to support the parameterization and validation of channel models on a reliable statistical basis. Omnidirectional path loss (PL) models can be derived directly from such data, and it is well suited for assessing results from ray tracing (RT) tools, which become increasingly important for channel modeling at higher frequencies [22,23]. They are capable of generating large data sets with unlimited dynamic range and can provide directional information to complement the measurements.

In this paper, data from three recent urban microcellular (UMi) measurement campaigns at 60 and 10 GHz is analyzed with focus on the PL and delay spread (DS). The frequencies were chosen since they represent sample points in the lower and the upper part of the range 6–100 GHz and hence are well suited to investigate frequency dependence. Moreover, a radio license could be obtained with sufficient bandwidth at 10 GHz, 60 GHz is an unlicensed band, and suitable measurement equipment was available. In view of complementing the measurements with validated simulation data, the results are compared with predictions from an in-house RT tool for the same environments. The findings provide valuable insight into the dependence of channel characteristics on the propagation scenario, distance and frequency. The presented modeling approaches and parameters were derived from several million channel observations for each scenario and can be used to refine 5G channel models and for comparative investigations. To the knowledge of the authors, a comparable unified analysis for the UMi channel at 60 and 10 GHz based on such large data sets has not been reported in the literature so far.

The measurement campaigns are briefly described in Section 2. Section 3 introduces the RT environment. The investigations on the PL and DS are presented in Section 4, including the comparison between simulation and measurement results. Conclusions are provided in Section 5.

## 2. Urban Microcellular Measurement Campaigns

To provide a data basis for model refinement, several measurement campaigns have been performed in Berlin at 60 GHz and 10 GHz. Two of them are related to UMi street canyon scenarios, namely “street canyon, city center” (SC-CC) [24] and “street canyon, residential area” (SC-RA) [24,25]. The third UMi scenario is “open square, city center” (OS-CC). Table 1 summarizes information on the environments and the collected data. The OS-CC measurement campaign is briefly described in Section 2.2.

### 2.1. Channel Sounder

All measurements were performed with the same setup, namely the in-house “High Performance Digital Radio Testbed” (HIRATE). Each device contains a commercial field-programmable gate array (FPGA) board, a self-developed converter board and several plug-on modules like filters, clocks and modulators. Two fully parallel signal chains allow dual-channel/frequency measurements [25].

The transmitter (TX) device generates a periodic baseband sounding signal based on a Frank sequence of length 256. The signal is up-converted to an intermediate frequency (IF) of 2.4 GHz. External mixers and filters are used to convert the IF signal to 10 and 60 GHz, respectively. The signal is finally fed into power amplifiers and radiated via the transmit antenna. The RX amplifies the received signal with a low noise amplifier. It is down-converted to IF and fed into the second HIRATE device, where it is converted to baseband. An internal trigger allows capturing snapshots of the signal with a high repetition rate. Most of the processing to compute the channel impulse response (CIR) is done via off-line post-processing, besides a real-time averaging procedure. It performs an $N_{\mathrm{avrg}}$-times waveform averaging before storing the digitized signal, improving the signal-to-noise ratio by an averaging gain of $G_{\mathrm{avrg}\left|\mathrm{dB}\right.}=10\cdot \log_{10}N_{\mathrm{avrg}}$. Synchronization between TX and RX is established via temperature-controlled rubidium clocks. The setup is calibrated on site via back-to-back measurement. Details on the HIRATE can be found in [26].

### 2.2. Open Square Measurements

**Figure 1:**
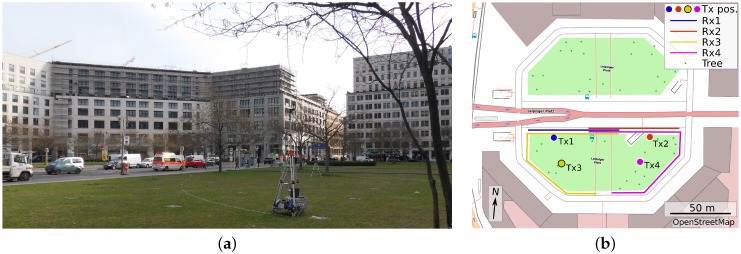
Measurement environment “open square, city center” (OS-CC): (**a**) photo with transmitter (TX) pole at position Tx3 and (**b**) map with TX positions and receiver (RX) tracks.

The OS-CC measurements at 60 GHz were performed on “Leipziger Platz”, which is an open square of octagonal shape and a diameter of approx. 150 m in the city center of Berlin. It is bordered by modern multi-story buildings, as can be seen in Figure 1a.

The TX of the channel sounder was placed at four different positions on the square. The antenna was mounted on a pole of 3.5 m height to simulate street furniture deployment. In accordance with the SC-CC and SC-RA measurements, the RX was mounted on a trolley at a height of 1.5 m and moved along the sidewalk at constant speed of 0.5 m/s. Figure 1b shows a map of the square with the TX positions and the RX tracks. For the majority of the RX positions, there was line-of-sight (LOS) between the TX and RX antenna. The measurement bandwidth was 250 MHz and a transmit power of 15 dBm could be achieved. Vertically polarized antennas with omnidirectional pattern in azimuth and 2 dBi gain were used at the TX and RX. A CIR snapshot took 65.5 μs and was recorded every 800 μs, corresponding to 0.4 mm spacing between adjacent RX positions. The overall data set acquired for the OS-CC scenario comprises 2.4 million CIRs.

## 3. Ray Tracing Simulations

**Figure 2:**
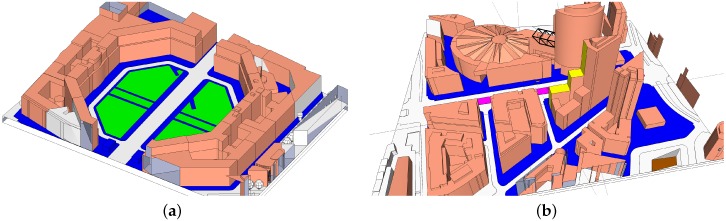
Top view of 3D models used for ray tracing. (**a**) OS-CC scenario; (**b**) “street canyon, city center” (SC-CC) scenario.

As support for the UMi measurements, RT simulations were performed for the OS-CC and SC-CC environments. The polarimetric RT tool, which has been developed by the Fraunhofer Heinrich Hertz Institute (Berlin, Germany) based on NVIDIA’s OptiX engine, makes use of parallelized processing on the graphics processing unit (GPU) [27]. It applies geometrical optics (GO) and the uniform theory of diffraction (UTD) for reflections on convex surfaces. The measurement environments are incorporated in form of 3D models from Leipziger Platz (Figure 2a) and Potsdamer Strasse (Figure 2b). They were manually extended to include street furniture and tree trunks [28].

All building surfaces were assumed to be glass with relative permittivity $\varepsilon_{r}=8.9-j0.13$ [29], where *j* is the imaginary unit. The ground was modeled with $\varepsilon_{r}=6.14-j0.30$, the value defined for concrete in [30], since the sidewalk is paved with concrete setts. The trees were assumed to be smooth, dielectric cylinders with $\varepsilon_{r}=1.57-j0.096$, the value for wood in [30]. The lamp posts were modeled as smooth and perfectly electrically conducting cylinders. Up to one transmission and four reflections were admitted. Isotropic, vertically polarized antennas were used, which has been found to well reflect the omnidirectional measurement antennas as long as only distances above 5 m are considered and their gain is subtracted. The RX measurement tracks with 0.4 mm spacing were fully re-simulated with the ray tracer. For each simulated RX point, a discrete equivalent baseband CIR was composed from the simulated rays based on their complex amplitude and propagation delay by binning (small bin size of 0.4 ns). Rays falling in one delay bin were added coherently. To provide fully comparable results, the simulated CIRs were band limited to the measurement bandwidth of 250 MHz, and the same post-processing was applied.

## 4. Evaluations

### 4.1. Power Delay Profiles

**Figure 3:**
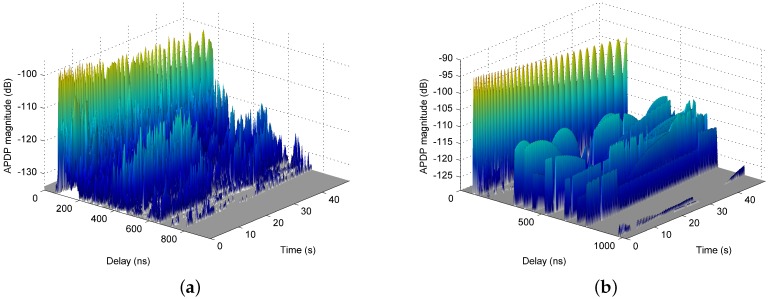
Evolution of averaged power delay profiles (APDPs) over time for TX position Tx1 and mobile RX on Track Rx1 from (**a**) measurement and (**b**) simulation.

Figure 3a shows a typical result of one measurement run on Track Rx1 in the form of averaged power delay profiles (APDPs) evolving over time. During the measurement run, the RX moved away from the TX, starting at 25 m distance and ending at 50 m. Each APDP has been obtained by averaging over K= 250 “instantaneous power delay profiles” $|h_{k}{\left(t\right)|}^{2}$ at consecutive RX points according to

(1) $$P\left(\tau \right)=\frac{1}{K}\sum_{k=1}^{K}{\left|h_{k}\left(\tau \right)\right|}^{2},$$

where P(τ) denotes the APDP and $h_{k}\left(\tau \right)$ is the *k*th CIR. K= 250 corresponds to a spatial averaging over 10 cm or 20 wavelengths. Comparative investigations with different factors have shown that the dominant effect of this kind of averaging on the given data is the reduction of thermal noise and phase noise. It is clearly visible that the first resolvable multipath component (MPC) is subject to strong fading. Based on geometric considerations, the distinct regular structure can clearly be attributed to the superposition of the direct path and the ground reflection, which cannot be resolved with 250 MHz bandwidth for larger distances. Further MPCs can be observed up to 800 ns delay. They are also subject to fading on a scale above 50 cm. However, overall, the APDP is relatively sparse. The MPCs will lead to time dispersion on the one hand. On the other hand, the related propagation paths can be very beneficial if the LOS path is blocked.

Figure 3b illustrates the corresponding simulated APDPs. The first MPC and its fading structure are well reproduced by the ray tracer and further strong MPCs are identified. However, in the measurements, they underlie a stronger fading on a time scale of several seconds. This indicates that the ray tracer is able to predict arriving path clusters, but not potential intra-cluster paths from a structured surface, e.g., outer walls with windows inwards. The granularity of the 3D model is of the order of 1 m and does not account for such structures. Furthermore, scattering, blockage and dynamic changes of the environment caused by cars and pedestrians cannot be considered. The ray tracer also tends to overestimate the strength of MPCs arriving with long delays, which gives rise to further investigations. The reason may be partially diffuse rather than ideal specular reflections. However, it is important to note that the results have been achieved without calibration of the ray tracer, but based on the simple assumptions mentioned above.

### 4.2. Path Loss

**Figure 4:**
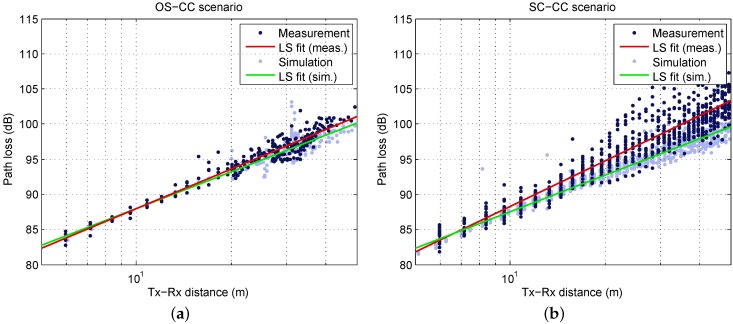
Measured and simulated path loss (PL) versus distance with linear regression line; (**a**) OS-CC scenario; (**b**) SC-CC scenario.

In this section, the local area PL is analyzed. To mitigate the strong fading of the first MPC as observed in Figure 3 and to eliminate a bandwidth dependence of the PL, a spatial averaging over at least one fading period must be performed [16]. In the present case, this could be achieved by choosing K= 3125, which corresponds to 1.25 m RX movement. The local area channel gain $G_{C}$ was calculated by integrating over the entire APDP and subtracting the estimated noise power $P_{N}$. For omnidirectional antennas, the antenna-deconvolved local area PL is then given by

(2) $$L_{\mathrm{PL}\left|\mathrm{dB}\right.}=-10\log_{10}G_{C}+G_{\mathrm{TX}\left|\mathrm{dB}\right.}+G_{\mathrm{RX}\left|\mathrm{dB}\right.},$$

where $G_{\mathrm{TX}}$ and $G_{\mathrm{RX}}$ are the antenna gains at the TX and the RX. Figure 4a illustrates the PL for the measurement runs with LOS (no blockage). As explained above, each data point results from 3125 CIRs. Data from distances below 5 m has been discarded, since the received power was significantly reduced by the elevation beamwidth of the antennas (at d=2m, the RX antenna was directly below the TX antenna). In addition to the calculated PL, the log-distance least-squares (LS) fit is shown. It relates to the well-known PL exponent model with a floating intercept point (IP) [31]:

(3) $$L_{\mathrm{PL}}{\left(d\right)}_{\left|\mathrm{dB}\right.}={\bar{L}}_{\mathrm{PL}}{\left(d_{0}\right)}_{\left|\mathrm{dB}\right.}+10n\log_{10}\left(\frac{d}{d_{0}}\right)+X_{\sigma },$$

where *d* denotes the TX–RX distance. The IP ${\bar{L}}_{\mathrm{PL}}{\left(d_{0}\right)}_{\left|\mathrm{dB}\right.}$ is the mean PL at the reference distance $d_{0}$, *n* is the PL exponent and $X_{\sigma }$ is a zero-mean Gaussian random variable (in dB) with standard deviation *σ*, which accounts for shadow fading.

The linear regression yields ${\bar{L}}_{\mathrm{PL}}\left(d_{0}\right)=82.3\;\mathrm{dB}$ for $d_{0}=5\;m$, n=1.88 and σ=1.03dB. Hence, the IP is very close to free space propagation (82.0 dB) and *n* is slightly below. The same evaluation is shown for the SC-CC scenario in Figure 4b. In this case, the IP is ${\bar{L}}_{\mathrm{PL}}\left(d_{0}\right)=81.9\;\mathrm{dB}$ for $d_{0}=5\;m$, and a PL exponent of n=2.13 is obtained. The shadow fading standard deviation is slightly higher, namely σ=2.03dB.

Table 2 summarizes the PL parameters for all measured UMi scenarios. The IP is given for the reference distance of $d_{0}=1\;m$ to be comparable with most of the literature values. Nevertheless, the models should not be applied outside the distance range, which has been used for the LS fit, namely the range where reliable measurement data is available without outage. It is denoted as validity range in Table 2. If for comparison reasons a different reference distance $\tilde{d_{0}}$ shall be used, the new IP can simply be derived from the model itself by calculating ${\bar{L}}_{\mathrm{PL}}{\left(\tilde{d_{0}}\right)}_{\left|\mathrm{dB}\right.}={\bar{L}}_{\mathrm{PL}}{\left(d_{0}\right)}_{\left|\mathrm{dB}\right.}+10n\log_{10}\left(\frac{\tilde{d_{0}}}{d_{0}}\right)$. *n* and *σ* are not affected as a matter of principle.

Under LOS conditions, *n* is basically close free-space propagation. A significantly smaller value (n=1.62) only occurs in the street canyon at 10 GHz. It can be caused by a waveguiding effect, which is not significant at 60 GHz. With 57.7 dB, the IP at $d_{0}=1\;m$ is significantly higher than the theoretical PL in free space (52.4 dB), which is an effect of the LS fit in connection with n<2. (Note that d=1m is beyond the validity range.)

As expected, the IP is larger for non-line-of-sight (NLOS), since the transition from LOS to NLOS results in a significant drop of the received power [16,32]. It is noteworthy that the NLOS scenario yields surprisingly low PL exponents. This, of course, is related to the scenario, but also because the LS fit has been limited to the distance range 5–80 m (propagation around one building corner only) to avoid outage at both 10 and 60 GHz to maintain full comparability. For the measured RA scenario, the PL exponent is higher for 60 GHz.

Figure 4 shows the simulated PL in addition to the measured results. For OS-CC, the simulation shows a good agreement with the measurement: the regression lines are very close to each other and the standard deviation is predicted very well (see Table 2). Discrepancies arise for the SC-CC scenario at larger distances: the simulations yield a lower PL exponent and a smaller *σ*. Making use of recorded videos during the measurements, investigations revealed that the LOS was temporarily obstructed by persons with increasing occurrence for increasing distance. Since pedestrians have not been included in the simulations, these effects cannot be reproduced. For the OS-CC scenario, obstruction was unlikely due to the TX positions on the lawn and the much lower density of pedestrians.

### 4.3. Delay Spread Statistics

To characterize the temporal spread of the channel, the root mean square (RMS) DS $\tau_{\mathrm{rms}}$ was calculated on the basis of APDPs according to

(4) $$\tau_{\mathrm{rms}}=\sqrt{\frac{\int_{0}^{\tau_{\max}}\tau^{2}P\left(\tau \right)\;d\tau }{\int_{0}^{\tau_{\max}}P\left(\tau \right)\;d\tau }-\tau_{m}^{2}},\;\tau_{m}=\frac{\int_{0}^{\tau_{\max}}\tau P\left(\tau \right)\;d\tau }{\int_{0}^{\tau_{\max}}P\left(\tau \right)\;d\tau },$$

where $\tau_{m}$ and $\tau_{\max}$ denote the mean and the maximum excess delay, respectively. A 25 dB threshold relative to the strongest MPC was used to exclude thermal noise and make the results fully comparable [32,33].

**Figure 5:**
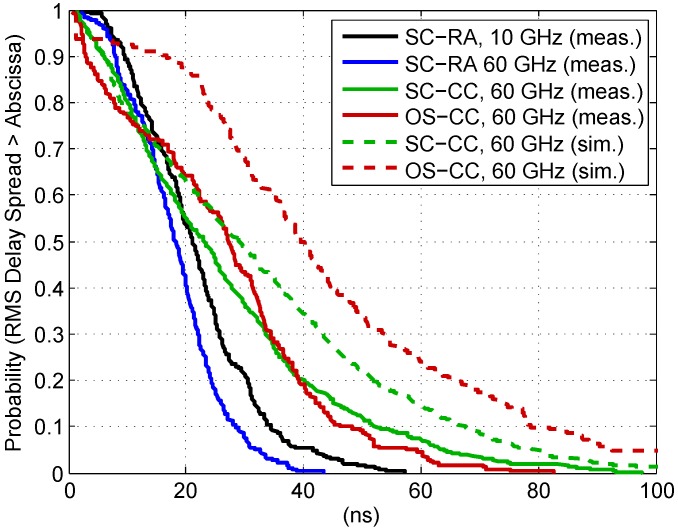
Empirical complementary cumulative distribution functions (CCDFs) of the root mean square (RMS) delay spread (DS) for all measured and simulated UMi line-of-sight (LOS) scenarios.

The empirical complementary cumulative distribution functions (CCDFs) of all measured and simulated UMi LOS scenarios are illustrated in Figure 5. They show a clear trend towards smaller DS for the SC-RA scenario. This is most probably related to the street canyon width, which is much smaller than for SC-CC environment and compared to the dimensions of the square (OS-CC). In addition, glass façades are dominant in the city center (OS-CC and SC-CC), which are more reflective than the plastered walls in the residential area (SC-RA). The difference in DS is more distinct for the peak values, which occur with lower probability. Table 3 summarizes the statistical parameters.

The 95%-quantile ($Q_{\mathrm{DS},0.95}$) is almost twice as large for SC-CC compared to SC-RA. Furthermore, it can be noticed that the SC-RA channel is slightly more dispersive at 10 GHz, which can be related to relative surface roughness and oxygen absorption at 60 GHz. The latter may already take effect, since the excess traveling distance of reflected paths can easily exceed 100 m. On the other hand, the DS for OS-CC and SC-CC is very similar—$\mu_{\mathrm{DS}}$ is almost equal. The slightly deformed shape of the CCDF for OS-CC might be due to the fact that—in contrast to SC-CC—the measurement points are not equally distributed over the distance range and overall less DS values are available.

In this context, it is noteworthy that the empirical statistical distribution of the DS (and other channel metrics) depends on the distribution of the measurement points over distances as long as a correlation between the respective metric and distance cannot be excluded. Simulations on system level might assume an equal distribution of users within a cell [10], resulting in a linear increase of the probability with respect to distance. It is therefore advisable to investigate a possible distance dependence and, if applicable, consider it in the channel model (see Section 4.4).

Equivalent evaluations were made with the RT data, available for SC-CC and OS-CC. To ensure full comparability, the CIRs were limited to 250 MHz bandwidth and the RMS DS $\tau_{\mathrm{rms}}$ was calculated on the basis of APDPs with the same threshold. The CCDFs are also shown in Figure 5. It can be seen that the ray tracer tends to overestimate the DS. The curves of the SC-CC scenario are still fairly close to each other, and the maximum DS is around 100 ns for the measurement as well as for the simulation. However, the deviation is larger for the OS-CC scenario. More detailed comparisons revealed that the RT predicts paths with relatively large delays, which do not exist in the measurement results, or whose power is larger (see also exemplary comparison in Figure 3).

It is important to note that the DS observed by an operational mm-wave system using directional antennas or antenna arrays with analog beamforming will be different from the reported values measured with omnidirectional antennas, since only a subset of propagation paths will be illuminated. However, the purpose of the presented evaluations is to quantitatively investigate the characteristics of the (antenna-decoupled) propagation channel on a fully comparable statistical basis. The results are valuable to parameterize and validate stochastic models and deterministic prediction tools, taking in turn into account directional information from the latter and from measurements e.g., with mechanical antenna steering. A directional channel model is then able to incorporate arbitrary antenna patters and multi-antenna configurations [14].

### 4.4. Distance Dependence of the Delay Spread

**Figure 6:**
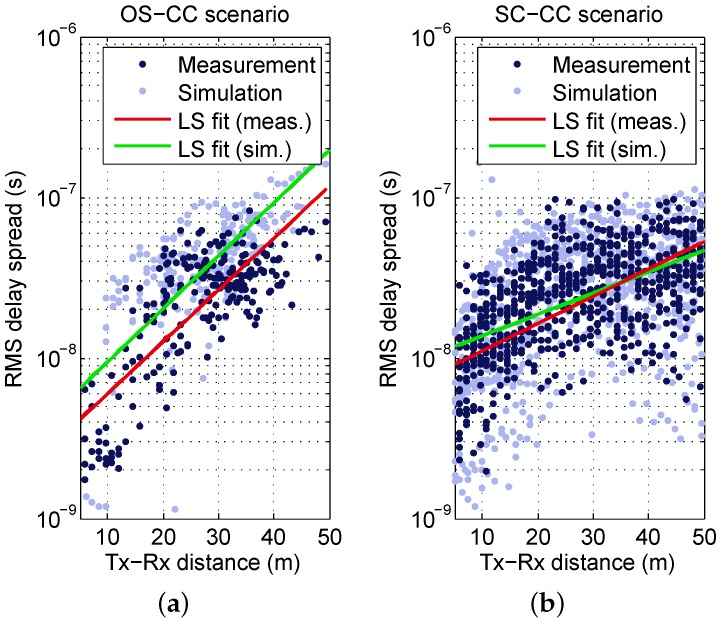
Measured and simulated DS as a function of distance with linear least-squares (LS) fit: (**a**) OS-CC scenario; (**b**) SC-CC scenario.

In this section, the dependence of the DS on distance is investigated in more detail. In Figure 6, the DS for the OS-CC and SC-CC scenarios is plotted (in logarithmic) scale against the distance. A distinct increase can be observed for the OS-CC scenario, which motivates the use of a linear regression model. It is expressed by

(5) $$\log_{10}\left(\frac{\tau_{\mathrm{rms}}}{1\;s}\right)\left(d\right)=\alpha +\beta d+\Gamma_{\epsilon },$$

where *α* is the IP at d=0m, *β* is the slope and $\Gamma_{\epsilon }$ is a zero-mean random variable with standard deviation *ϵ*. The values obtained by LS fitting are given in Table 4.

Figure 6b illustrates the same evaluation for the SC-CC environment, where more samples are available. The DS values similarly increase as a function of distance, though the slope is less steep and a saturation effect can be observed at around 20 m. Hence, the model should not be extrapolated to distances beyond the validity range. It shall be noted that the saturation is not related to an exclusion of values due to an insufficient dynamic range of the APDPs, but the evaluable dynamic range is always large enough.

Figure 6 also includes the DS derived from simulations—the corresponding parameters for the distance-dependent model are given in Table 4. As already seen in Section 4.3, the prediction yields a noticeably higher DS than the measurement for the OS-CC scenario. However, according to Figure 6a, the behavior of the DS over distance is very similar and the linear regressions are parallel. This is also reflected by the model parameters. Whereas the slope *β* of the simulations fits the measurements, the IP *α* has a slight difference that matches the offset between the regressions. The larger standard deviation is mainly caused by some data points close to $10^{-9}$, where no significant multipaths were predicted. For the SC-CC scenario, see Figure 6b, the simulated DS matches the measured DS very well. The ray tracer reproduces the behavior over a distance in a good manner, and also saturates around 100 ns. Smaller DS in the simulation are caused by non-predicted paths, which increases the standard deviation in Table 4. *α* is in agreement with the measurements. The slope *β* varies slightly, which is also obvious in Figure 6.

**Figure 7:**
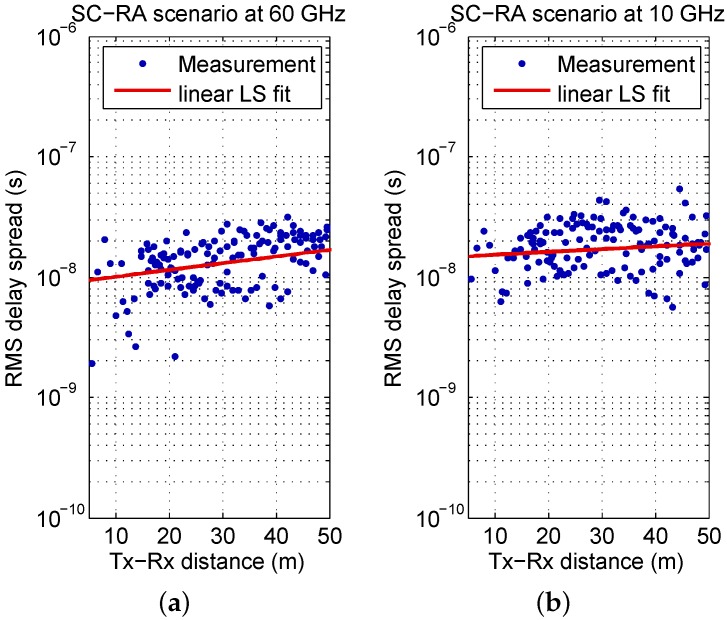
Measured DS as a function of distance with linear LS fit for “street canyon, residential area” (SC-RA) scenario: (**a**) at 60 GHz; (**b**) at 10 GHz.

The measurement results for the SC-RA scenario are depicted in Figure 7. The gradient for 60 GHz is moderate (compared to OS-CC and SC-CC). Nevertheless, it is clear that limiting the set to DS values attributed to distances below 50 m and calculating the empirical CCDF on this basis, would further increase the difference to the OS CC and SC-CC curves in Figure 5. For 10 GHz, the gradient is very small—the calculated DS is almost independent from distance.

Overall, it can be concluded that the RMS DS tendentially increases with distance for all measured UMi scenarios and the distance dependence is well predicted by the ray tracer. The potential of a steeper gradient is increased for propagation environments that yield high DS values.

## 5. Conclusions

The availability of reliable and accurate channel models is one of the crucial aspects for supporting the deployment of ultra-high capacity 5G networks. Proposed models that target a frequency range between 0.5 and 100 GHz and corresponding parameter tables still require further refinement and substantiation by statistically reliable measurement data in multiple frequency bands and various key scenarios.

In this paper, the results of three UMi measurement campaigns performed in Berlin, Germany, have been presented, focusing on the PL and DS of the UMi access channel at 60 and 10 GHz. The evaluations are based on several million channel observations for each scenario, and full comparability has been ensured by uniform data processing. The findings aim to support the validation and improvement of current 5G channel models above 6 GHz. It revealed that the ground reflection has a strong impact on the LOS channel. Since its superposition with the LOS path results in a distinct regular fading of the first resolvable MPC, it will largely affect the instantaneous received power and channel conditions for a mobile user in a quasi-deterministic manner. State-of-the-art GSCMs do not consider this behavior, and it is strongly recommended to further investigate and incorporate this effect.

At 60 GHz, the PL exponents under LOS conditions were found to be close to free-space propagation. A smaller value, namely 1.62, has been observed for 10 GHz in the narrow street canyon. The RMS DS shows a clear dependence on the scenario and its dimensions. Under LOS conditions, it is largest for the open square, and the values for the wide street canyon with modern buildings exceed the results obtained in the residential area. The DS is slightly larger at 10 GHz (data for narrow street canyon only) compared to 60 GHz for LOS, indicating only a weak frequency dependence. For NLOS, however, the difference is significant for the given scenario (median values are 38 and 16 ns for 10 and 60 GHz, respectively). The DS tendentially increases with distance for all measured UMi scenarios, but saturates around 100 ns. The distance dependence is strong for the open square scenario and the wide street in the city center, but less distinct in the narrow street canyon with residential buildings. It is well predicted by complementary RT simulations, although not all channel characteristics could be reproduced with satisfying accuracy. It is important to underpin such simulation results by measurements and to further improve the prediction tools.

## Figures and Tables

**Table 1:** Summary of measured urban microcellular (UMi) scenarios and main parameters.

Scenario	OS-CC	SC-CC [24]	SC-RA [25]
Site	Berlin city center,	Berlin city center,	Berlin residential area
	Leipziger Platz	Potsdamer Str.	Kreuzberg
Dimensions	Square diameter: ≈150 m	Street canyon width: ≈52 m	Street canyon width: 15–20 m
Frequency	60.0 GHz	60.0 GHz	60.4 GHz and 10.0 GHz
Bandwidth	250 MHz	250 MHz	250 MHz
Antenna type (TX and RX)	omni, vertical pol.	omni, vertical pol.	omni, vertical pol.
HPBW in elevation	≈80 deg	≈80 deg	≈80 deg at 60.4 GHz,
			≈60 deg at 10.0 GHz
Antenna height (TX/RX)	3.5 m/1.5 m	3.5 m/1.5 m	5.0/1.5 m
Conditions	mainly LOS	mainly LOS	LOS/NLOS
Distance	2–50 m	2–50 m	4–210 m
Spacing between meas.	0.4 mm	0.4 mm	0.4 mm
points for mobile RX						
Number of collected CIRs	2.4 million	6.4 million	6.3 million

**Table 2:** Path loss (PL) parameters derived from measurements for all UMi scenarios and values from ray tracing simulations for “open square, city center” (OS-CC) and “street canyon, city center” (SC-CC) scenario.

Scenario	Freq. (GHz)	Conditions	Measurement			Simulation			Valid. Range (m)
			${\bar{L}}_{\mathrm{PL}}\left(1\;m\right)$	*n* (dB)	*σ* (dB)	${\bar{L}}_{\mathrm{PL}}\left(1\;m\right)$ (dB)	*n*	*σ* (dB)		
OS-CC	60.0	LOS	69.2	1.88	1.03	69.2	1.74	1.17	5–50
SC-CC	60.0	LOS	67.0	2.13	2.03	69.2	1.72	0.97	5–50
SC-RA	60.4	LOS	67.0	2.07	2.53	-	-	-	10–210
		NLOS	69.7	2.67	4.93	-	-	-	10–80
	10.0	LOS	57.7	1.62	2.95	-	-	-	10–210
		NLOS	61.7	2.10	7.36	-	-	-	10–80

**Table 3:** Statistical parameters of the root mean square (RMS) delay spread (DS): mean $\mu_{\mathrm{DS}}$, standard deviation $\sigma_{\mathrm{DS}}$, median $m_{\mathrm{DS}}$ and 95%-quantile $Q_{\mathrm{DS},0.95}$.

Scenario	Frequency (GHz)	Conditions	$\mu_{\mathrm{DS}}$ (ns)	$m_{\mathrm{DS}}$ (ns)	$\sigma_{\mathrm{DS}}$ (ns)	$Q_{\mathrm{DS},0.95}$ (ns)
OS-CC	60.0	LOS	26.5	27.3	16.9	59.5
SC-CC	60.0	LOS	26.6	23.2	19.1	63.9
SC-RA	60.4	LOS	18.3	18.2	7.84	32.5
		NLOS	20.9	15.9	13.4	43.8
	10.0	LOS	21.9	21.1	10.2	42.2
		NLOS	37.7	38.2	17.4	67.9

**Table 4:** Parameters for distance-dependent DS model for line-of-sight (LOS): intercept point *α*, slope *β* and standard deviation *ϵ*.

Scenario	Freq. (GHz)	Measurement			Simulation			Valid. Range (m)
		*α*	*β*	*ϵ*	*α*	*β*	*ϵ*	
OS-CC	60.0	−8.54	0.0322	0.262	−8.35	0.0329	0.306	5–50
SC-CC	60.0	−8.13	0.0170	0.287	−8.06	0.0135	0.371	5–50
SC-RA	60.4	−8.05	0.0055	0.185	-	-	-	5–50
	10.0	−7.84	0.0024	0.216	-	-	-	5–50

## Abbreviations

The following abbreviations are used in this manuscript:

3D	three-dimensional
3GPP	3rd Generation Partnership Project
5G	fifth generation
APDP	averaged power delay profile
CCDF	complementary cumulative distribution function
CIR	channel impulse response
deg	angular degree
DS	delay spread
FPGA	field-programmable gate array
GO	geometrical optics
GPU	graphics processing unit
GSCM	geometry-based stochastic channel model
HIRATE	High Performance Digital Radio Testbed
HPBW	half-power beamwidth
IF	intermediate frequency
IP	intercept point
ITU-R	International Telecommunication Union Radiocommunication Sector
LOS	line-of-sight
LS	least-squares
METIS	Mobile and Wireless Communications Enablers for the Twenty-twenty Information Society
MiWEBA	Millimetre-Wave Evolution for Backhaul and Access
mm-wave	millimeter-wave
MPC	multipath component
NLOS	non-line-of-sight
omni	omnidirectional
OS-CC	open square, city center
PL	path loss
pol.	polarization
RMS	root mean square
RT	ray tracing
RX	receiver
SC-CC	street canyon, city center
SC-RA	street canyon, residential area
TX	transmitter
UMi	urban microcellular
UTD	uniform theory of diffraction
